# 32A9, a novel human antibody for designing an immunotoxin and CAR-T cells against glypican-3 in hepatocellular carcinoma

**DOI:** 10.1186/s12967-020-02462-1

**Published:** 2020-08-03

**Authors:** Xiaoyu Liu, Fang Gao, Longwei Jiang, Meng Jia, Lei Ao, Ming Lu, Liming Gou, Mitchell Ho, Shaochang Jia, Fei Chen, Wei Gao

**Affiliations:** 1 Key Laboratory of Human Functional Genomics of Jiangsu Province, School of Basic Medical Sciences, Nanjing Medical University, Nanjing, Jiangsu 211166 People’s Republic of China; 2 Department of Biotherapy, Nanjing Jinling Hospital, Nanjing, Jiangsu 210002 People’s Republic of China; 3 School of Chemistry and Molecular Biosciences, University of Queensland, Brisbane St Lucia, QLD 4072 Australia; 4 Laboratory of Molecular Biology, Center for Cancer Research, National Cancer Institute, National Institutes of Health, Bethesda, MD 20892 USA; 5 Department of Medical Oncology, Jilin Cancer Hospital, Changchun, Jilin 130012 People’s Republic of China

**Keywords:** Liver cancer, Glypican-3, Antibody, Immunotoxin, Chimeric antigen receptor

## Abstract

**Background:**

Treatment of hepatocellular carcinoma (HCC) using antibody-based targeted therapies, such as antibody conjugates and chimeric antigen receptor T (CAR-T) cell therapy, shows potent antitumor efficacy. Glypican-3 (GPC3) is an emerging HCC therapeutic target; therefore, antibodies against GPC3 would be useful tools for developing immunotherapies for HCC.

**Methods:**

We isolated a novel human monoclonal antibody, 32A9, by phage display technology. We determined specificity, affinity, epitope and anti-tumor activity of 32A9, and developed 32A9-based immunotherapy technologies for evaluating the potency of HCC treatment in vitro or in vivo.

**Results:**

32A9 recognized human GPC3 with potent affinity and specificity. The epitope of 32A9 was located in the region of the GPC3 protein core close to the modification sites of the HS chain and outside of the Wnt-binding site of GPC3. The 32A9 antibody significantly inhibited HCC xenograft tumor growth in vivo. We then pursued two 32A9-based immunotherapeutic strategies by constructing an immunotoxin and CAR-T cells. The 32A9 immunotoxin exhibited specific cytotoxicity to GPC3-positive cancer cells, while 32A9 CAR-T cells efficiently eliminated GPC3-positive HCC cells in vitro and caused HCC xenograft tumor regressions in vivo.

**Conclusions:**

Our study provides a rationale for 32A9 as a promising GPC3-specific antibody candidate for HCC immunotherapy.

## Background

Hepatocellular carcinoma (HCC) accounts for the majority of primary liver cancer cases and is the sixth most common and the second most lethal cancer worldwide [1, 2]. The majority of HCC occurs in patients with underlying chronic liver disease, mostly as a result of hepatitis B or hepatitis C virus infection and alcohol consumption [3]. However, the lack of efficient treatment has led to the highest rate of increase in both new cases and mortality of HCC among all tumors in the past decade [4].

Glypican-3 (GPC3) is a heparan sulfate proteoglycan that is anchored on the cell surface by glycophosphatidylinositol (GPI) [5–7]. GPC3 is expressed in fetal liver, where it is involved in organ morphogenesis by regulating cell proliferation through modulation of Wnt and Hedgehog signaling, and is turned off upon physiological maturity [8–10]. However, the expression of GPC3 is restored in most HCC patients through unknown mechanisms. GPC3 is specifically expressed in 70–80% of HCC patients but not in normal adult tissues [11]. Moreover, the expression level of GPC3 is correlated with poor prognosis of HCC [12–14]. Many studies have reported that GPC3 promotes the development of HCC as a coreceptor in Wnt and HGF signaling [15–18]. Therefore, GPC3 is identified as a potent diagnostic marker and promising therapeutic target for HCC.

The mature form of human GPC3 has a protein core consisting of seven disulfide bonds that are highly conserved in the glypican family. In the flexible C-terminal region, GPC3 contains two binding sites (S495 and S509) for heparan sulfate (HS) chains [19]. Our previous data demonstrate that the protein core and the HS chains of GPC3 both contribute to the activation of Wnt signaling [20–23]. We identified one Wnt-binding motif on the GPC3 protein core [21, 23], and the another on the HS chains of GPC3 [20, 22], then we isolated two human monoclonal antibodies, HN3 [23, 24] and HS20 [18, 20, 22], to target these Wnt-binding motifs, respectively. HN3 and HS20 exhibited significant blocking effects on Wnt signaling and inhibited HCC tumor growth in vitro and in vivo [22–24]. These results indicate that targeting GPC3 with functional blocking antibodies would be a feasible strategy for HCC therapy.

In the present study, we were interested in developing a novel anti-GPC3 monoclonal antibody that would recognize an epitope outside of the Wnt-binding motif or known epitope of other GPC3 antibodies and would be used to construct more effective antibody-based immunotherapies, such as immunotoxins [24, 25] and CAR-T cells [26, 27]. We obtained a human monoclonal antibody (32A9) against GPC3 by phage display technology. 32A9 recognized a region of the GPC3 protein core close to the modification sites of the HS chain. We made a human IgG antibody, an immunotoxin and CAR-T cells based on the 32A9 scFv. These three treatments all showed potent antitumor activity by in vitro or in vivo evaluations. Altogether, our findings suggested that 32A9, a non-Wnt blocking antibody, was a potent tool for GPC3-specific targeted therapy for HCC.

## Methods

### Clinical samples and cell lines

The normal liver tissue and HCC tumor tissue specimens used in the current study were obtained from the patients recruited from the Nanjing Drum Tower Hospital (Nanjing, Jiangsu) between November 2018 and March 2019. This study was approved by the Ethical Committee of Nanjing Drum Tower Hospital, and every patient provided written informed consent. Huh-7 cells were a kind gift from Dr. Xin-Wei Wang at the National Cancer Institute (Bethesda, MD). A431, SK-hep-1, Hep3B, HepG2 and HEK293T cells were purchased from American Type Culture Collection (Manassas, VA). All cells were cultured in DMEM (HyClone, Logan, UT) supplemented with 10% fetal bovine serum (VACCA, St. Louis, MO), 100 U/ml penicillin, and 0.1 mg/ml streptomycin (HyClone, Logan, UT) and were incubated in 5% CO_2_ at 37 °C. A431 cells were engineered to highly express GPC3 by transfection with a plasmid encoding full-length GPC3. PBMCs were isolated by Ficoll density centrifugation of healthy donors’ blood, obtained under the ethically protocol approved by Nanjing Jinling Hospital (Nanjing, Jiangsu). The isolated PBMCs cultured in RPMI (HyClone, Logan, UT) supplemented with 10% fetal bovine serum (VACCA, Murphysboro, IL), 100 U/ml penicillin, and 0.1 mg/ml streptomycin and were incubated in 5% CO_2_ at 37 °C. All cell lines were evaluated and validated by their morphology and growth rate. All the cell lines were confirmed to be free of mycoplasma contamination by PCR using specific primers.

### Plasmids, proteins, and antibodies

The sequence of full-length GPC3 was cloned into the pLVX vector. For protein purification, truncated GPC3 (Q25-S550) was fused to a human Fc tag and cloned into the pFUSE vector. All point mutants of GPC3 and the GPC3 mutant lacking the HS chains (GPC3ΔHS) were generated by introducing point mutations using the overlapping PCR method. The mFrizzled8 ECD-hFc plasmid was purchased from Addgene (Cambridge, MA). The 32A9 scFv sequence was fused to the human Fc tag and cloned into the pFUSE vector as well. The 32A9 heavy chain variable region and light chain variable region sequences were amplified by adding IL-2 signal peptide and were inserted into the expression vectors pFUSE-CHIg-HG1 and pFUSE2-CLIg-hk (Invivogen, San Diego, CA), respectively. All plasmids were identified by sequencing and then transfected into 293T cells. After collecting the supernatant, protein purification was accomplished with a protein A affinity column (GE Healthcare, Milwaukee, WI). GPC3-his and GPC5-his proteins were purchased from R&D (Minneapolis, MN).

YP7, a control mouse monoclonal antibody against GPC3 [28], was used to evaluate GPC3 expression in immunohistochemistry staining, flow cytometry and ELISA assays.

### Phage display

The TG1 clone was picked and cultured overnight in 2YT medium at 37 °C. GPC3-Fc protein in PBS buffer was used to coat an ELISA plate, which was incubated overnight at 4 °C. The next day, the phage library (Tomlison I library) and the coated wells were blocked with PBST with 3% milk at room temperature for 1 h. Then, the blocked phages were added to the coated wells and incubated at room temperature for 1 h. After washing 20 times with PBST, the GPC3-binding phages were eluted with 100 mM triethylamine (Sigma-Aldrich, Xuhui, Shanghai). All eluted phages were collected and used to infect TG1 cells. After incubation with helper phages, the eluted phages were rescued with a titer of approximately 10^11^ ~ 10^12^ pFU/ml for the next round of panning.

### Structural modeling

The human GPC3 (NCBI Gene ID: 2719), mouse GPC3 (NCBI Gene ID: 14734), and human GPC5 (NCBI Gene ID: 2262) sequences were submitted to the protein structure modeling web server SWISS-MODEL [29]. The C-terminal region was submitted to the full-chain protein structure prediction server Robetta [30]. The full-length GPC3 model was constructed by joining the GPC3 protein core model and the C-terminal region model according to the GPC1 topology structure and small angle X-ray scattering (SAXS) data [31]. The 32A9 scFv model was obtained by an antibody modeling tool (Prediction of ImmunoGlobulin Structure, PIGS) [32]. All homolog template sequence and structure file (GPC1 PDB ID: 4YWT) were selected and downloaded from the RCSB protein data bank [33]. ZDOCK SERVER was used to predict the epitope of 32A9 [34]. PyMOL was used to analyze and render structure models.

### Elisa

GPC3-hFc protein (5 μg/ml) was used to coat ELISA wells at 4 °C overnight. The wells were blocked with PBS containing 3% milk for 0.5 h at 37 °C. The antibodies or phage were added to the wells and incubated at 37 °C for 0.5 h. After washing with PBST 3 times, goat anti-human kappa chain HRP antibody (for 32A9) (Life Tech, Peoria, IL), goat anti-mouse HRP antibody (for YP7) (Jackson ImmunoResearch, West Grove, PA) or rabbit anti-M13 HRP antibody (for phage) (GE Healthcare, Milwaukee, WI) was added to the wells and incubated at 37 °C for 0.5 h. TMB and H_2_SO_4_ were added to detect the OD_450nm_ value.

### Flow cytometry

A single**-**cell suspension was incubated with 5 µg/ml of the indicated antibodies for 1 h on ice and then incubated with a 1:200 dilution of anti-human PE antibody (for 32A9) (Thermo, Pudong New Area, Shanghai) or anti-mouse PE antibody (for YP7) (Thermo, Pudong New Area, Shanghai) for 1 h on ice. The cells were analyzed using FACS Calibur (BD Biosciences, San Jose, CA).

### Immunotoxin purification

The sequence of 32A9 scFv was fused to a truncated and deimmunized Pseudomonas exotoxin (mPE24) fragment [35–37] and then cloned into the pMH212 vector [37]. BL21 competent cells (Weidi Biotechnology, Minhang District, Shanghai) were transfected with the 32A9-mPE24 plasmid and induced with 1 mM IPTG for 1.5 h. The inclusion body was collected, washed and lysed by lysozyme to obtain the raw extraction of recombinant protein. The recombinant protein was denatured, refolded, and buffer exchanged by overnight dialysis. The refolded recombinant protein was acquired by chromatography with ÄKTA pure (GE Healthcare, Milwaukee, WI).

### Cytotoxicity of immunotoxin

A total of 10^4^ cells were seeded into 96-well plates and cultured overnight at 80–90% density, and then different concentrations of immunotoxin were added to the wells. After 72 h, the OD_450 nm_ values were detected by using a CCK-8 kit (Beyotime, Songjiang, Shanghai) to analyze the cytotoxicity of immunotoxin.

### Generation of 32A9 CAR-T cells

A 32A9-based second generation 4-1BB CAR fragment was synthesized (GenScript, Nanjing, Jiangsu). To detect the expression of CAR, an EGFP coding sequence was added upstream of the 32A9-CAR fragment separated by an F2A sequence. The whole sequence was subcloned into the pLVX lentiviral vector. The same version of FMC63 4-1BB CAR targeting CD19 was used as a negative control [38]. To produce viral supernatant, HEK293T cells were cotransfected with CAR lentiviral vector, packaging plasmid and envelope plasmid using Lipofectamine 2000 according to the manufacturer’s protocol. The supernatant was collected and concentrated 72 h later. PBMCs were stimulated for 24 h with anti-CD3/anti-CD28 antibody-coated beads (Invitrogen, Carlsbad, CA) at a 2:1 bead-to-T cell ratio in growth medium supplemented with 50 U/ml IL-2 (GenScript, Nanjing, Jiangsu). Activated T cells were then transduced with the lentivirus expressing CARs (MOI = 10). Cells were counted and fed fresh growth medium every other day. The transduction efficiency was detected by GFP fluorescence with flow cytometry.

### In vitro CAR-T cell function assays

CAR-T cells were cocultured with GPC3-expressing cancer cells at the indicated ratios for 18 h. Then, the supernatant was collected to measure cytotoxicity by an LDH release assay (Promega, Madison, WI), and IL-2 release was measured by an IL-2 detection kit (MultiSciences, Hangzhou, Zhejiang).

### Animal tests

NYG mice which were established by knocking out Prkdc and Il2rg in NOD mice by Crispr-Cas9 technology. Four- to six-week-old female NYG mice were purchased from the Animal Facility of Nanjing Medical University. All mice were treated under the protocol approved by the Ethics Committee of the Animal Core Facility of Nanjing Medical University. To evaluate the antitumor activity of the 32A9 antibody, five million Huh-7 cells were subcutaneously inoculated into five-week-old NYG mice. When the average tumor size reached ~ 50 mm^3^, the mice were treated with PBS or 10 mg/kg 32A9 antibody by i.v. injection. Tumor dimensions were determined using calipers, and tumor volume was calculated by the formula V = 1/2 ab^2^, where a and b represent tumor length and width, respectively. To evaluate the antitumor activity of 32A9 CAR-T cells, five million of Huh-7 cells or luciferase-labeled Hep3B cells were subcutaneously inoculated into five-week-old NYG mice. When the average tumor size was ~ 50 mm^3^, 10 million PBMCs or 32A9 CAR-T cells were infused to mice for Huh-7 groups, PBS or 10 million FMC63-CAR-T cells or 32A9 CAR-T cells were infuse into mice for Hep3B groups by i.v. injection. The tumor size was measured every other day or imaged after the mice were injected with 100 µl D-luciferin (Yeasen, Pudong New Area, Shanghai) and imaged 10 min later. Living Image software was used to analyze the bioluminescence signal flux for each mouse as photons per second per square centimeter per steradian (photons/s/cm2/sr). Mice were killed when they showed any sign of sickness or when the tumor burden reached 1500 mm^3^ or the bioluminescence signal reached 1 × 10^11^ photons/s/cm2/sr.

### Statistical analysis

Representative results were obtained with at least three independent experiments. All group data (except those indicated) are expressed as the mean ± standard deviation (SD) of a representative experiment performed at least in triplicate, and similar results were obtained in at least three independent experiments. All statistical analyses were conducted using GraphPad Prism 5.0. Two-tailed Student’s *t* test of the means and two-way ANOVA were used for statistical analysis, with P* < 0.05 defined as significant.

## Results

### GPC3 was highly expressed in HCC patients and HCC cell lines

**Fig. 1:**
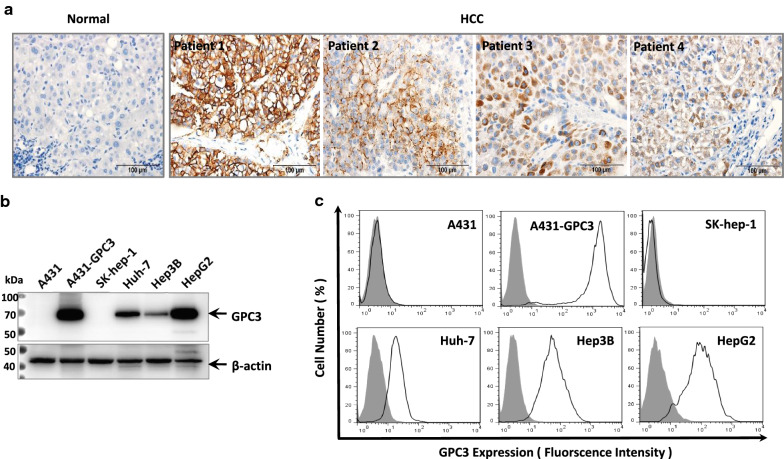
GPC3 expression in HCC patient tissue and HCC cell lines. **a**. Immunohistochemistry staining of GPC3 in HCC patient tumor tissues and normal liver tissues. Scale bar: 100 μm. **b** Western blot to detect GPC3 expression in HCC cells. **c** Flow cytometry analysis to detect GPC3 cell surface expression in the indicated cells. Filled curve: Isotype control staining. Open curve: anti-GPC3 staining. A431 and A431-GPC3 are set up as GPC3-negative cell line control and GPC3-positive cell line control for GPC3 staining, respectively

To evaluate the expression of GPC3 in HCC patients, we performed immunohistochemistry staining of HCC tumors and normal liver tissue (Table 1). We found that GPC3 was specifically expressed in HCC tumors but not in normal liver tissue. Some of the HCC patients exhibited obvious cell surface staining of GPC3, whereas others exhibited less (Fig. 1a). Western blot showed that GPC3 was highly expressed in HCC cell lines (Hep3B and Huh-7), as well as in HepG2, a hepatoblastoma cell line (Fig. 1b). We also detected the cell surface expression of GPC3 in these cells. A431-GPC3, Huh-7, Hep3B and HepG2 cells showed obvious cell surface expression of GPC3, but not in A431 and Sk-hep-1 cells (Fig. 1c). Altogether, GPC3 was specifically expressed in HCC and would be a promising target due to its cell surface expression.

**Table 1:** Characteristics of HCC patients

Case	Gender	Age	Edmondson grade	Tumor size (cm)	Tumor multiplicity	Vascular invasion	Satellite nodule	Metastasis
#1	Male	60	II	2.8	1	Absent	0	Absent
#2	Male	50	II	8	1	Absent	0	Absent
#3	Male	61	II or III	3.8	1	Absent	1	Absent
#4	Male	66	II	3	1	Absent	0	Absent

*TNM* tumor-node-metastasis

### Isolation of the human monoclonal antibody 32A9 against GPC3

**Fig. 2:**
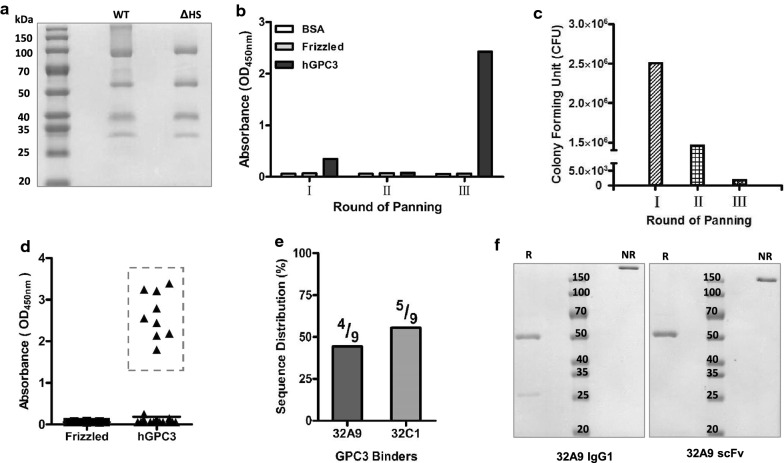
Antibody screening by phage display. **a** SDS-PAGE of purified wild-type GPC3 (WT) and mutant GPC3 (∆HS) hFc fusion proteins. **b** Polyclonal phage ELISA to detect the antigen binding activity of three rounds of rescued phages. BSA and frizzled are used as negative antigen controls. **c** Eluted phage numbers of three rounds of panning. **d** Monoclonal phage ELISA to analyze GPC3-specific binder. Nine positive clones are marked in dashed boxes (n = 192 clones). BSA and frizzled are used as negative antigen controls. E. Sequence analysis of 9 positive binders identified in **d**. **f** SDS-PAGE of purified 32A9 scFv-Fc and 32A9 IgG. *R* :reducing, *NR*: non-reducing

To obtain a new anti-GPC3 human antibody, we first produced recombinant GPC3-Fc protein and GPC3ΔHS-Fc protein in human HEK293T cells as antigens for screening (Fig. 2a). Then, we screened the phage library by three rounds of panning. The signal of GPC3-specific binders increased dramatically (Fig. 2b), although number of colony forming units decreased during each round of panning (Fig. 2c). After panning, 192 clones were selected randomly for further evaluation. Among them, we selected 9 positive clones with an OD_450 nm_ value at least ten-fold higher than that of the negative control for sequencing (Fig. 2d). Two sequences (32A9 and 32C1) were finally identified (Fig. 2e). We then constructed vectors to purify these two antibodies in both an scFv-Fc format and IgG format. Unfortunately, clone 32C1 lost its binding activity to GPC3 when expressed in eukaryotic cells; therefore, we only focused on 32A9 scFv-Fc and 32A9 IgG in our study (Fig. 2f).

**Fig. 3:**
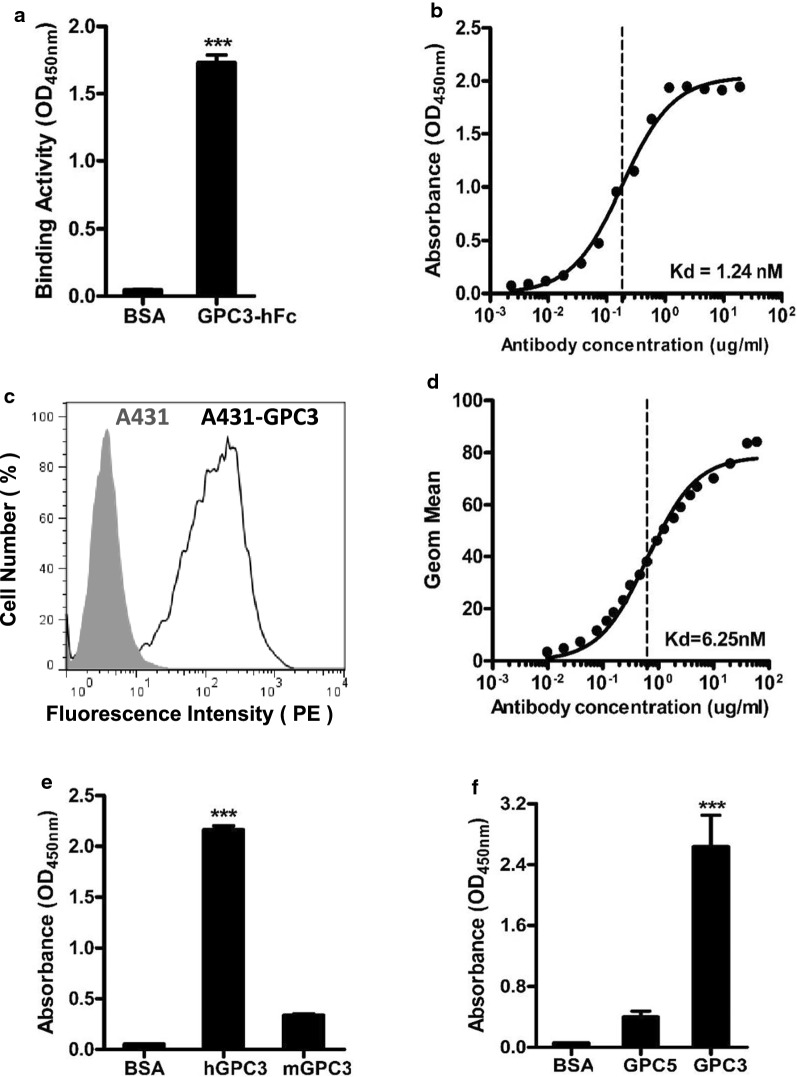
Binding properties of the 32A9 IgG antibody. ELISA to detect 32A9 binding activity (**a**) and affinity (**b**) to GPC3-hFc protein. Flow cytometry to detect the 32A9 binding signal (**c**) and affinity (**d**) to GPC3-positive cells. **e** ELISA to detect 32A9 binding specificity to human GPC3 and mouse GPC3. **f** ELISA to detect 32A9 binding specificity to GPC3 and GPC5. Values represent the mean ± SD, with ***p < 0.001

To further characterize the 32A9 antibody, we first detected its binding activity to the GPC3-Fc protein. The 32A9 antibody showed strong binding activity (Fig. 3a) with an affinity of approximately 1.24 nM (Fig. 3b). We next measured the binding activity of 32A9 to GPC3-positive cells. The 32A9 antibody specifically bound to A431-GPC3 cells but not A431 cells (Fig. 3c). The binding affinity was approximately 6.25 nM (Fig. 3d). To evaluate the specificity of the 32A9 antibody to GPC3 from different species, we compared 32A9 binding to human GPC3 and mouse GPC3. We found that 32A9 antibody had poor binding activity to mouse GPC3 (Fig. 3e). Moreover, we selected human GPC5, which had the most sequence identity with human GPC3 among the glypican family, to test whether 32A9 antibody had cross reactivity with other glypicans. We found that 32A9 antibody could not recognize GPC5 well (Fig. 3f). Overall, 32A9 antibody had promising binding activity and specificity to human GPC3.

## 32A9 antibody recognized the middle region of the GPC3 protein core

**Fig. 4:**
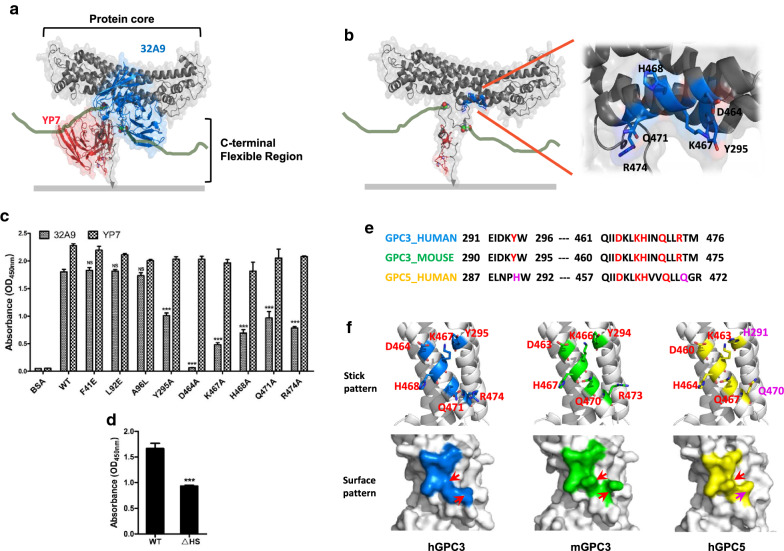
Epitope mapping of 32A9. **a** Structural model of the 32A9/GPC3 complex. YP7 is used as a control antibody against GPC3. GPC3: gray. 32A9: blue. YP7: red. HS chains: green. **b** The predicted epitope of 32A9. Essential residues of the 32A9 epitope are labeled in blue. The region recognized by YP7 is labeled in red. HS chain binding residues (S495 and S505) are shown as atoms. **c** ELISA to detect the binding activity of 32A9 and YP7 on the indicated mutant GPC3. Point mutations Y295A, D464A, K467A, H468A, Q471A and R474A were constructed to destroy the 32A9 epitope, whereas point mutations F41E, L92E and A96L are used as control residues. Values represent the mean ± SD, with p*** < 0.001. **d** ELISA to detect 32A9 binding activity on wild-type GPC3 and GPC3ΔHS. Values represent the mean ± SD, with ***p < 0.001. **e** Sequence comparison of human GPC3 epitope and its corresponding sequences in mouse GPC3 and human GPC5. The key residues of 32A9 epitope are showed in red. Two different residues in human GPC5 are labeled in pink. **f** Structural comparison of 32A9 epitope among modeled human GPC3, mouse GPC3 and human GPC5. Arrows indicate the residues with different orientation of side chain

To identify the epitope of the 32A9 antibody, we first generated a structural model of the GPC3 protein core using the GPC1 structure as a template in the homology modeling tool SWISS MODEL and obtained the model of the C-terminal region of GPC3 by the ab initio modeling tool Robetta. The model of full-length GPC3 was constructed by joining the two models of GPC3 according to the GPC1 topology structure and small angle X-ray scattering (SAXS) data [31]. The 32A9 scFv model was acquired by an antibody modeling tool (Prediction of ImmunoGlobulin Structure, PIGS). Then, we predicted the interactions in the 32A9/GPC3 complex by ZDOCK SERVER, an automatic protein docking server for analyzing protein–protein interactions. The predicted results showed that 32A9 bound to the middle region of the protein core of GPC3 (Fig. 4a). We then generated six point mutations on Y295, D464, K467, H468, Q471, and R474 according to the prediction (Fig. 4b). After obtaining these purified mutant GPC3 proteins, we detected the binding activity of the 32A9 antibody to them. As we predicted, these mutant GPC3 proteins were poorly recognized by the 32A9 antibody, whereas other mutations (F41E, L92E, and A96L) outside this region did not affect the binding of the 32A9 antibody. We also used YP7 as a control antibody targeting the C-terminus of GPC3 [28] to verify our results. Since none of these mutations were located in its epitope within residues 510─560, YP7 showed similar binding activity to these mutant GPC3 compared to wild-type GPC3 (Fig. 4c). Interestingly, we found that the 32A9 bound to GPC3 was quite close to the sites linked to HS chains according to our predicted model of the GPC3/32A9 complex. We then compared 32A9 antibody binding to wild-type GPC3 and GPC3ΔHS. The results showed that although GPC3ΔHS could also be recognized by 32A9, the binding activity was significantly reduced compared to that to wild-type GPC3 (Fig. 4d). These observations suggested that 32A9 recognized the middle region of the GPC3 protein core and that the HS chain of GPC3 also contributed to 32A9 binding. We also compared the epitope sequence of human GPC3 and its corresponding sequences in mouse GPC3 and human GPC5. Interestingly, even though mouse GPC3 exhibited exact the same sequence identity with human GPC3 in this region (Fig. 4e), but its conformation seemed more closer to human GPC5 (Fig. 4f). This information suggested that 32A9 recognized a unique conformational epitope on human GPC3 which determined its specificity.

## 32A9 antibody and immunotoxin showed anti-tumor potency

**Fig. 5:**
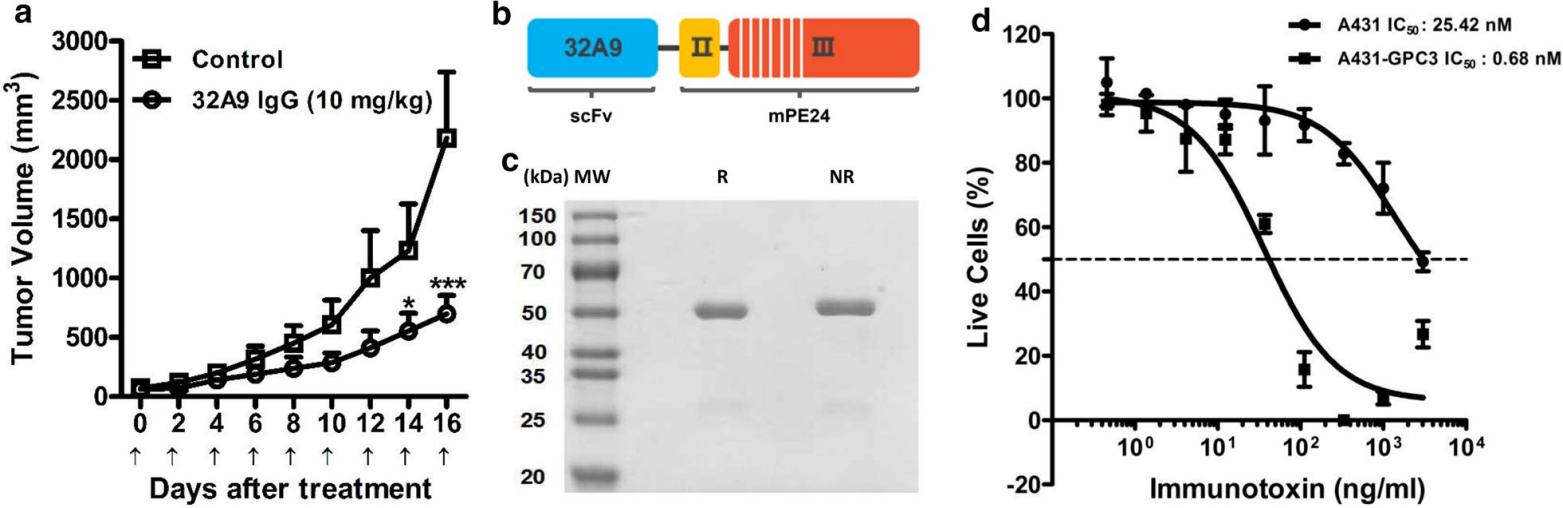
Anti-tumor effects of 32A9 IgG and 32A9 immunotoxin. **a** Anti-tumor activity of 32A9 IgG in NYG mice with Huh-7 xenograft tumors. Mice are treated with 32A9 IgG (10 mg/kg) or PBS every other day. Arrows indicate antibody injection. Values represent the mean ± SE, with *p < 0.05 and ***p < 0.001. **b** Schematic diagram of the 32A9 immunotoxin (32A9-mPE24). *PE*: Pseudomonas exotoxin A. **c** SDS-PAGE to show the purified 32A9-mPE24. *R*: reducing. *NR*: non-reducing. **d** WST assay to detect the cytotoxicity of 32A9-mPE24. The dashed line indicates the IC_50_ value. Values represent the mean ± SD

To evaluate the anti-tumor activity of the 32A9 antibody in vivo, we inoculated NYG mice with Huh-7 cells. Then, the mice were treated with 10 mg/kg 32A9 antibody or the same volume of PBS by tail vein injection every other day. 32A9 antibody treatment did not significantly affect the body weight of the mice. Two weeks after treatment, the tumor volumes of the 32A9 antibody-treated group were significantly smaller than those of the control group (Fig. 5a), indicating the potent anti-tumor activity of 32A9 as a naked antibody. An immunotoxin is an antibody-toxin conjugate that can be internalized into tumor cells and efficiently kill tumor cells by inhibiting protein synthesis after binding to the cell surface tumor antigen [25]. To investigate the possibility of using 32A9 as a suitable antibody for constructing an effective immunotoxin, we fused 32A9 scFv to the truncated and deimmunized Pseudomonas exotoxin (mPE24) [37] to constructed 32A9-mPE24, a new immunotoxin against GPC3 (Fig. 5b) and purified it by chromatography (Fig. 5c). We then treated the cells with 32A9-mPE24 to determine its cytotoxicity in vitro. Our results showed that 32A9-mPE24 selectively inhibited the growth of GPC3-positive cancer cells with an IC_50_ value of 0.68 nM but had no obvious effect on GPC3-negative cells (Fig. 5d). Overall, 32A9 exhibited anti-tumor potency as a naked antibody and an immunotoxin.

## 32A9 CAR-T cells significantly inhibited HCC tumor growth in vitro and in vivo

**Fig. 6:**
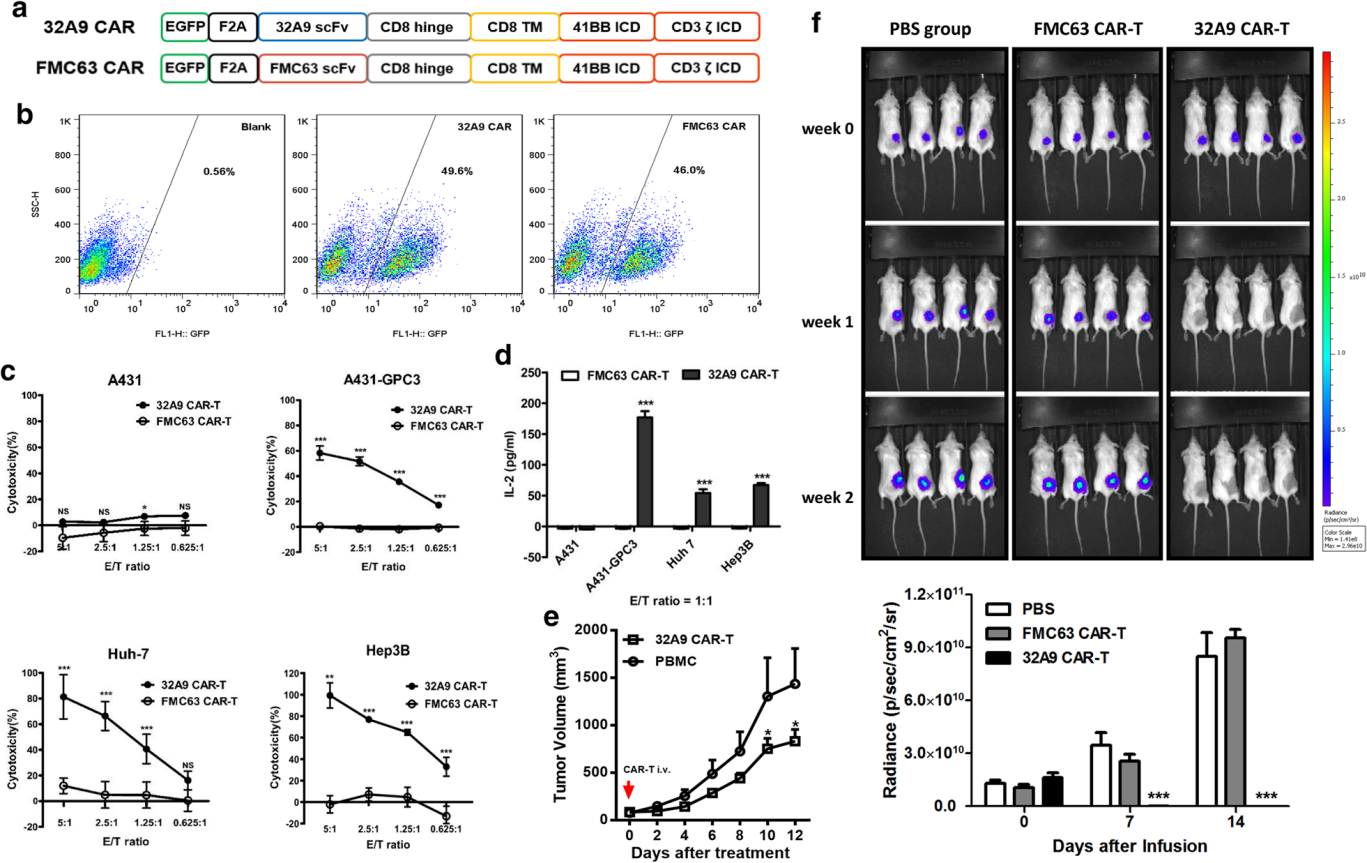
Anti-tumor activity of 32A9 CAR-T cells. **a** Schematic diagram of 32A9 CAR in the second generation 4-1BB format. FMC63 CAR is used as a negative control. **b** Transduction efficiency of 32A9 CAR-T cells and FMC63 CAR-T cells. **c** LDH release assay to detect the cytotoxicity of 32A9 CAR-T cells. Values represent the mean ± SD, with *p < 0.05 and ***p < 0.001. **d** ELISA to detect the activation of 32A9 CAR-T cells by measuring IL-2 release. Values represent the mean ± SD, with ***p < 0.001. **e***In vivo* animal test of 32A9 CAR-T cells on Huh-7 xenograft tumors. Ten million PBMCs or 32A9 CAR-T cells are infused into mice by tail vein injection. The tumor size is measured every other day. Values represent the mean ± SEM, with *p < 0.05. **f***In vivo* animal test of 32A9 CAR-T cells on Hep3B xenograft tumors. PBS or ten million of FMC63 CAR-T cells or 32A9 CAR-T cells were infused into mice carrying Hep3B xenograft tumors by tail vein injection once a week for 2 weeks. The tumor burdens were quantified by measuring luciferase fluorescence weekly. Values represent the mean ± SEM, with ***p < 0.001

To evaluate whether 32A9 could be further used for immune cell-targeted therapy, we constructed second generation 4-1BB CAR-T cells based on 32A9. An anti-CD19 CAR (FMC63 CAR) [38] was used as our negative control (Fig. 6a). Overall, the transduction efficiency of CAR-T cells was around 50% (Fig. 6b). We then detected the in vitro cytotoxicity of 32A9 CAR-T cells. We found that 32A9 CAR-T cells could efficiently lyse GPC3-positive cells (A431-GPC3, Huh-7 and Hep3B cells), but FMC63 CAR-T cells did not have any effect on these cells (Fig. 6c). Similar results were also observed in the IL-2 production assay: 32A9 CAR-T cells were activated significantly when co-cultured with GPC3-positive HCC cells (Fig. 6d). We next performed an in vivo animal study to further verify the potential anti-tumor effect of 32A9 CAR-T cells. 32A9 CAR-T cells induced dramatic tumor regression when injected into mice with Huh-7 xenograft tumors (Fig. 6e) and Hep3B xenograft tumors (Fig. 6f). Taken together, our results indicate that 32A9 is a promising antibody candidate for designing CAR-T cells for HCC therapy.

## Discussion

The HCC-specific expression and important function of GPC3 as a Wnt coreceptor make GPC3 an attractive therapeutic target in HCC. Here, we reported that 32A9, a novel human monoclonal antibody against GPC3, would satisfy the requirements for anti-GPC3 immunotherapy.

Many GPC3-specific antibodies have been developed for HCC diagnosis or therapy; however, clear epitope information is available for few of them [39]. Except for the HS chain-specific antibody [18, 20, 22], GPC3 antibodies with determined epitopes could be roughly divided into two groups. One group is generated by immunizing mice with the C-terminal flexible region of GPC3 (residues 510–560), where has the least sequence identity among different species and would have better immunogenicity in mice. 1G12 [11], GC33 [40] and YP7 [28] are representative antibodies currently reported. These antibodies all showed ideal affinity and have been widely used for HCC diagnosis [41]. Furthermore, GC33 and YP7 are being developed for anti-GPC3 cancer therapies including CAR-T cells, bispecific T-cell engagers and ADCs [42–44]. The other group includes HN3 [23], a unique single-domain human antibody isolated by phage display targeting the Wnt-binding region on the N-lobe of GPC3 protein core [21]. HN3 exhibits a Wnt-specific blocking effect as predicted and would be a useful tool to investigate the detailed interaction of Wnt and GPC3 [21, 23, 24]. In the current study, we obtained 32A9, an antibody targeting an epitope outside of the Wnt-binding region of the GPC3 protein core. Therefore, 32A9 would not compete with HN3 that binds N-lobe of GPC3 or any antibodies (e.g., YP7, GC33) recognizing the C-terminal epitope. This property suggests that 32A9 could be coupled with the proper anti-GPC3 antibody to develop ELISA detection reagents.

Because the epitope of 32A9 identified in our study is quite close to the HS binding sites (residues S495 and S509), it seems that the HS chains of GPC3 contribute to the binding of 32A9 (Fig. 4e). Therefore, we suspect that 32A9 would influence Wnt signaling by affecting the function of HS chains, which also contribute to GPC3-mediated Wnt activation. Unfortunately, when we performed the Wnt reporter assay, 32A9 did not show an obvious inhibitory effect on Wnt activation. Since we determined that 32A9 bound to the middle region but not to the Wnt-binding motif of the GPC3 protein core [21] or HS chains [20], we believe that 32A9 might not influence the GPC3-mediated activation of Wnt signaling, either through the protein core or HS chains. Interestingly, it has been reported that GPC3 could also interact with frizzled though its HS chains to induce a synergistic stimulation of Wnt signaling [9, 21]. However, how and where the HS chains interact with frizzled is still unclear. We could not exclude the possibility that 32A9 might influence the interaction of the HS chain and frizzled. Therefore, it will be worth investigating whether 32A9 affects frizzled-dependent Wnt activation in our future studies.

In the present study, we constructed a human IgG, an immunotoxin and CAR-T cells as representative formats to verify the multiple potential applications of 32A9. The 32A9 immunotoxin showed specific cytotoxicity on GPC3-positive cells, which indicates that 32A9 could also be used to design other internalization-dependent antibody drugs, such as antibody drug conjugates [44]. 32A9 CAR-T cells showed a significant effect on eliminating HCC tumors, suggesting the feasibility of 32A9 CAR-NK cells [45] or other similar engineered immune cell therapies [46]. Bispecific antibodies normally form a complicated interaction between tumor cells, antibodies and T cells, which requires the antibody to maintain the antigen-binding property rigorously when coupled with an anti-CD3 antibody fragment [42, 47]. Although 32A9 IgG was evaluated in our study, whether 32A9 would also be functional when modified into a bispecific antibody will be evaluated later.

## Conclusions

Overall, we developed a novel human monoclonal anti-GPC3 antibody, 32A9, which targets the middle region of the GPC3 protein core. 32A9 IgG and 32A9-based immunotherapies showed potent antitumor activity. Our findings provide a rationale for 32A9 as a promising candidate antibody for GPC3 biological function investigation, HCC diagnosis and therapeutic applications.

## Data Availability

The datasets used and/or analyzed during the current study are available from the corresponding author on reasonable request.

## Abbreviations

HCC: Hepatocellular carcinoma
GPC3: Glypican-3
GPI: Glycophosphatidylinositol
CDR: Complementarity-determining region
CAR: Chimeric antigen receptor
